# Identification of Shared Pathways and Molecules Between Type 2 Diabetes and Lung Adenocarcinoma and the Impact of High Glucose Environment on Lung Adenocarcinoma

**DOI:** 10.1155/ije/7734237

**Published:** 2025-02-26

**Authors:** Mengsi Yang, Jianmin Luo, Yunna Zheng, Qunqing Chen

**Affiliations:** ^1^Department of Thoracic Surgery, Zhujiang Hospital of Southern Medical University, Guangzhou 510282, Guangdong Province, China; ^2^Department of Thoracic Surgery, Affiliated Hospital, Zhanjiang Medical University, Zhanjiang 524001, Guangdong Province, China; ^3^Department of Respiratory Medicine, Nanfang Hospital, Southern Medical University, Guangzhou 510515, Guangdong Province, China

**Keywords:** Alzheimer's disease, functional enrichment analysis, hub gene, LUAD, protein–protein interaction network, Type 2 diabetes mellitus, WGCNA

## Abstract

**Objective:** This research focused on exploring the shared pathophysiological bases of lung adenocarcinoma (LUAD) and Type 2 diabetes mellitus (T2DM).

**Methods:** The investigation into the molecular similarities between LUAD and T2DM involved querying the Gene Expression Omnibus for pertinent data. Upon pinpointing genes exhibiting differential expression, pathway enrichment analyses were executed to discern the molecular pathways shared by both conditions. In addition, GeneMANIA was employed to establish a protein interaction network, pinpointing STK26 as a critical gene. In addition, the influence of STK26 on the immune environment of the tumor was examined using tools such as the Microenvironment Cell Populations–counter to assess levels of stromal and immune cells in cancer tissues from expression profiles. Furthermore, a lung cancer cell model enriched in glucose was developed to facilitate the knockdown of STK26 using small interfering RNA. The influence of STK26 on A549 cell functionality was assessed using CCK-8, wound healing (scratch), and colony formation (cloning) assays.

**Results:** This will help ensure accuracy and relevance in the revised version. TGF-*β*, HIF-1, AGE–RAGE, extracellular matrix (ECM) components and function regulation, and cell adhesion were activated in LUAD and T2DM. WGCNA identified two main modules in LUAD, three main modules in T2DM, and 44 shared genes. ClueGO and GeneMANIA analyses focused on pathways regulating cell growth and mitosis. Our analysis revealed STK26 as a central gene that exhibits elevated expression levels in tissues affected by LUAD. Elevated expression of STK26 correlates with a diminished prognosis for LUAD patients. In patients with LUAD characterized by elevated STK26 levels, gene set enrichment analysis identified a notable upregulation in numerous metabolic pathways. These include glycolysis–gluconeogenesis, oxidative phosphorylation, and the conversion pathways between pentose and glucuronic acid, as well as the pentose phosphate pathway. Gene set variation analysis suggested that a high STK26 expression was related to glycolysis, hypoxia, MYC, oxidative phosphorylation, cell cycle, and citric acid cycle pathways. In the group exhibiting elevated levels of STK26, a marked upregulation of glycolytic pathway genes, including HK2, RPIA, IDH3G, and SORD, was noted. This upregulation indicates a correlation between STK26 expression and these pivotal glycolytic genes. MCP–counter analysis suggested that the group with a high STK26 expression level had reduced immune infiltration. Laboratory studies have demonstrated that LUAD cells thrive in a high-glucose setting, where STK26 expression notably surpasses that observed under standard conditions. In addition, suppressing STK26 using siRNA significantly curtails both the growth and movement of LUAD cells.

**Conclusion:** The research established a shared pathogenic basis between LUAD and T2DM. TGF-*β*, HIF-1, AGE–RAGE, ECM components and function regulation, cell adhesion, and additional signaling pathways are intricately linked with the pathophysiological mechanisms underlying both LUAD and T2DM. Thus, STK26 may affect the development of LUAD and T2DM by regulating glucose metabolism. Suppressing STK26 in a glucose-rich setting curtailed both the expansion and mobility of LUAD cells.

## 1. Introduction

Globally, lung cancer remains a principal cause of mortality due to cancer, among which lung adenocarcinoma (LUAD) is the predominant form [1]. Throughout the years from 2010 to 2014, even as lung cancer treatment methods improved, the five-year survival rate for individuals diagnosed with LUAD consistently ranged from 10% to 20% in different countries [2]. Type 2 diabetes mellitus (T2DM) is recognized globally as a chronic ailment [3]. With the aging of the global population, there is an annual increase in the cases of lung cancer diagnoses. In 2017, global estimates indicated that around 451 million individuals were living with diabetes. In individuals diagnosed with non-small cell lung cancer (NSCLC), approximately 8.7% were concurrently managing T2DM [4, 5]. T2DM as a metabolic disease has become increasingly common, and this disease can cause serious complications [6]. Research shows that T2DM increases susceptibility to various cancers, such as those of the colorectal, breast, gallbladder, endometrium, liver, and pancreas [7–9]. Increasing evidence suggests an association between LUAD and T2DM. Several studies have reported an elevated risk and poor prognosis of LUAD in patients with T2DM. However, the underlying mechanisms that link LUAD and T2DM remain unclear.

Time and again, epidemiological studies have pinpointed diabetes as a key risk factor in the onset of LUAD. Current research underscores the significant impact of concurrent health conditions on the prognosis outcomes in patients with NSCLC [4]. Diabetes, a prevalent chronic condition, appears to interact complexly with LUAD. A comprehensive meta-analysis analyzing 17 distinct studies, which collectively included over one million individuals, determined that diabetes significantly increases the likelihood of being diagnosed with LUAD compared to nondiabetic individuals [10]. The risk is even higher in certain populations, such as women and Asians. Recent research has elucidated multiple mechanisms linking T2DM with LUAD. Research highlights the PI3K/Akt kinase and Ras/MAP kinase pathways. In addition, elevated insulin levels frequently lead to increased hydrogen peroxide production, exacerbating oxidative stress and furthering genetic damage in tumor cells. Moreover, the persistent inflammation typically associated with diabetes can facilitate the proliferation and dissemination of cancer cells. However, treatments for diabetes such as metformin may have protective effects against LUAD. Metformin has anticancer properties as it inhibits tumor growth and reduces inflammation [11]. Numerous observational studies suggest a link between metformin use and a reduced risk of developing LUAD, though findings across studies vary.

Thus, while the correlation between diabetes and LUAD is becoming more apparent, the underlying biological mechanisms are intricate and likely involve several factors. Further research is essential to elucidate the link between these conditions and to develop effective preventive and therapeutic strategies. It is crucial for medical professionals to recognize this correlation and diligently observe patients with T2DM for indications of LUAD.

## 2. Materials and Methods

### 2.1. Data Acquisition

We conducted our research by extracting gene expression profiles from the Gene Expression Omnibus (GEO) database, available at https://www.ncbi.nlm.nih.gov/geo/. Specific search parameters, “type 2 diabetes mellitus” and “non-small cell lung cancer,” were used to select data relating exclusively to patients diagnosed with both T2DM and LUAD. We evaluated the retrieved datasets based on specific criteria, ensuring that each dataset contained profile information for both case and control groups. Second, each sample originated exclusively from lung tissue. Third, none of the pancreatic cancer (PC) cases underwent chemoradiotherapy prior to surgical removal. Fourth, it was imperative that these datasets included raw data suitable for further analysis. Ultimately, for advanced research, GEO datasets GSE76896, GSE43458, GSE25724, GSE20966, GSE31210, and GSE68465 were chosen. Data concerning somatic mutations and transcriptomes were retrieved from The Cancer Genome Atlas (TCGA), accessed via https://portal.gdc.cancer.gov/.

### 2.2. Differentially Expressed Genes (DEGs) Were Analyzed Using Gene Ontology (GO) and Kyoto Encyclopedia of Genes and Genomes (KEGG) Pathway Tools

To elucidate the biological functions and interactions of DEGs associated with T2DM and LUAD, annotations were carried out through GO and KEGG pathway analyses. Prior to employing the limma R package for differential gene analysis, quantitative normalization of data across various samples was conducted. DEGs showing a *p* value below 0.05 were selected for detailed analysis. In the analysis of T2DM and LUAD, DEGs exhibiting an absolute log fold change exceeding 0.5 and 1, respectively, were emphasized. A significance threshold was established at *p* values less than 0.05. Analysis and visualization of the GO and KEGG data were conducted using the “clusterProfiler” and “GOplot” R packages [12].

### 2.3. The Study Utilized the Weighted Gene Coexpression Network Analysis (WGCNA) Approach

This algorithm groups genes into modules according to their expression similarities and analyzes the connections between these modules and different biological traits [13]. Our research employed the “WGCNA” R package to construct coexpression networks, concentrating on 6000 genes exhibiting the highest median absolute deviation, relevant to T2DM and LUAD. We first established an adjacency matrix, setting a soft threshold β at 7 for T2DM and 9 for LUAD. This was combined with a gene–gene correlation matrix to measure node connectivity. Following this transformation, we used hierarchical clustering to construct a dendrogram that clearly defined the coexpression modules among the genes. The final steps involved computing the module eigengene (ME) and assessing its correlation with clinical traits, aiming to pinpoint modules associated with clinical outcomes.

### 2.4. Hub Genes Within Critical Modules Were Identified and Validated

Shared genes exhibiting positive correlation within the T2DM and LUAD modules were analyzed for overlap using the JVENN tool [14]. To categorize functions and visualize them, the ClueGO plugin for Cytoscape was used. It arranged nonredundant GO terms and illustrated a network of functionally interconnected gene clusters. This approach enabled an in-depth analysis of common genes, leveraging ClueGO to clarify their biological functions. For GO terms, a significance level was established with *p* values required to be less than 0.05. A protein–protein interaction (PPI) network, designed via the GeneMANIA prediction server, facilitated the identification of shared gene markers between T2DM and LUAD. This server synthesizes biological networks, aiding in the prioritization of genes and forecasting their functions. In addition, GO was employed to enrich hub clusters further.

### 2.5. Validation of Shared Genes in T2DM and LUAD

We validated hub-shared genes between T2DM and LUAD by analyzing DEGs in datasets GSE25724 (6 T2DM samples and 7 normal), GSE20966 (10 T2DM samples and 10 normal), GSE31210 (226 tumors and 20 normal), and GSE68465 (443 tumors and 19 normal) utilizing the R package “limma.” To depict the commonality of shared genes across test and validation cohorts, we employed the JVENN tool to create a Venn diagram illustrating the intersections of DEGs.

### 2.6. Gene Set Enrichment Analysis (GSEA) Was Conducted in Conjunction With Gene Set Variation Analysis (GSVA)

In LUAD cases, patients were categorized based on whether their expression of STK26 was above or below the median level. The GSEA approach generates enrichment scores (ESs) for gene clusters classified by function, facilitating the identification of distinct functional phenotypes [15]. GSEA analyzed the variance in biological pathways across the two groups, considering only those KEGG pathways significant with a false discovery rate below 0.05. GSVA, a refined approach for decoding gene expression data, evaluates the activity of gene sets linked to particular biological pathways or processes in individual samples. By annotating the gene expression matrix and using known gene sets such as GO terms and KEGG pathways, this approach quantifies the enrichment levels of gene sets within individual samples and exposes the general activity patterns of biological processes across varying samples. The output from GSVA is structured as a matrix, with each sample corresponding to a row and each gene set to a column. This format displays the ESs for each gene set across individual samples.

### 2.7. Assessment of the Immune Landscape

This approach modifies the traditional GSEA framework to generate specific ESs for individual samples [16]. The GSVA algorithm is designed to reformat a single-gene expression matrix into one focused on specific gene sets, from which it calculates ES [17]. By utilizing ssGSEA scores, comparisons were drawn regarding the quantity of immune cells and the functionality related to immunity between groups with high and low expressions of STK26. The ESTIMATE method, an acronym for the estimation of stromal and immune cells in malignant tumor tissues using expression data, was applied to calculate stromal and immune scores based on expression data within tumor tissues [18]. Spearman's correlation analysis was employed to explore the association between STK26 expression and immune cell presence, focusing on the expression patterns of genes associated with immune functions. This tool uses a pre-established gene expression signature file containing genes associated with different types of immune cells, such as specific markers of various immune cell subsets. The MCP–counter method estimates the quantities of various immune cell types in a tumor sample by analyzing gene expression data from tumor tissues.

### 2.8. Cell Culture and Transfection

A549 cells were propagated in Dulbecco's modified eagle medium supplemented with 10% fetal bovine serum, incubated at 37°C in an atmosphere containing 5% CO2. Functional experiments exclusively employed cells that were in the logarithmic phase of growth. To suppress STK26 expression, cells underwent transfection using specific STK26 siRNAs with the aid of Lipofectamine® 2000 (Invitrogen, United States of America), following the guidelines provided by the manufacturer. The siRNA sequences used were 5′-CUUAAACAGCAGGACGAGAAU-3′ for STK26-1 and 5′-AUUCUCGUCCUGCUGUUUAA-3′ for siSTK26-2. To verify the effect of a high glucose (HG) environment on LUAD cells, we treated LUAD cell line A549 with 25 mmol of HG medium and 5.5 mmol of normal medium, respectively, *in vitro*.

### 2.9. Extraction of RNA and Subsequent Real-Time Quantitative Polymerase Chain Reaction (qRT-PCR)

RNA extraction from the cells was carried out employing TRIzol reagent (Invitrogen, United States of America). This was followed by cDNA synthesis using the HiScript Synthesis kit (Vazyme, China). qRT-PCR analyses were conducted using a StepOnePlus system (Applied Biosystems, Foster City, California, United States of America) with Fast SYBR Green Master Mix (Roche). The qRT-PCR employed primers specific for STK26, with sequences forward, 5′ CAGAAGGACACAGTGATGATG′ and reverse, 5′ ACAACTCAGGGTTTGCACAA′. The 18s was used as the internal parameter primer, with sequences forward, 5′ CCTGGATACCGCAGCTAGGA′ and reverse, 5′ GCGGCGCAATACGAATGCCCC′. It was used as an endogenous control to normalize for differences in the amount of total RNA in each sample. The results were expressed relative to the control condition, which was arbitrarily assigned a value of 1.

### 2.10. Cell Counting Kit-8 (CCK-8) and Colony Formation

We assessed the growth potential of A549 cells through CCK-8 assays and colony formation evaluations. Utilizing the CCK-8 (C0038, Beyotime, China), we quantified growth rates by measuring absorbance at 450 nm. Measurements were taken 2 hours after incubation, employing an enzyme-linked immunosorbent assay technique at several designated time intervals. In the procedure assessing colony formation, each well of a six-well plate received around 1000 cells. After allowing for colony growth, the assay involved fixing the cells with 4% methanol and then applying a crystal violet stain to evaluate the development of colonies.

### 2.11. Wound Healing Assay

This setup facilitated the observation of monolayer cell growth using an inverted microscope. The cells were exchanged after treatment. A sterile micropipette tip (20 μL) was used to scratch the cell plate, with the tip held against the horizontal line behind the ruler. Using Image-Pro Plus software (Version 6.0), the scratch width for cells in each group was recorded at consistent time intervals. To quantify cell migration, the narrowing of the scratch was tracked from the initial time point and recorded periodically from 0 h. This change in width was then expressed as a percentage relative to the scratch's original width. This calculation served to quantify the cellular movement and migration capabilities within each group. Statistical analysis was then applied to the data collected.

### 2.12. Statistical Analysis

Employing R software (Version 4.1.0), statistical evaluations were conducted. To assess the disparities in gene expression between the tumor and adjacent normal tissues, the Wilcoxon test was utilized. The Kaplan–Meier method was applied to analyze survival rates across various groups, utilizing the log-rank test for comparative assessment. Multivariate Cox regression analysis was employed to evaluate the influence of STK26 expression on overall survival. The data of qPCR, scratch test, and CCK-8 experiments are presented as the mean ± standard error of the mean of three repeats, and the unpaired *t*-test was selected to comparethe two groups.

## 3. Results

### 3.1. Identification and Functional Enrichment of DEGs

**Figure 1:**
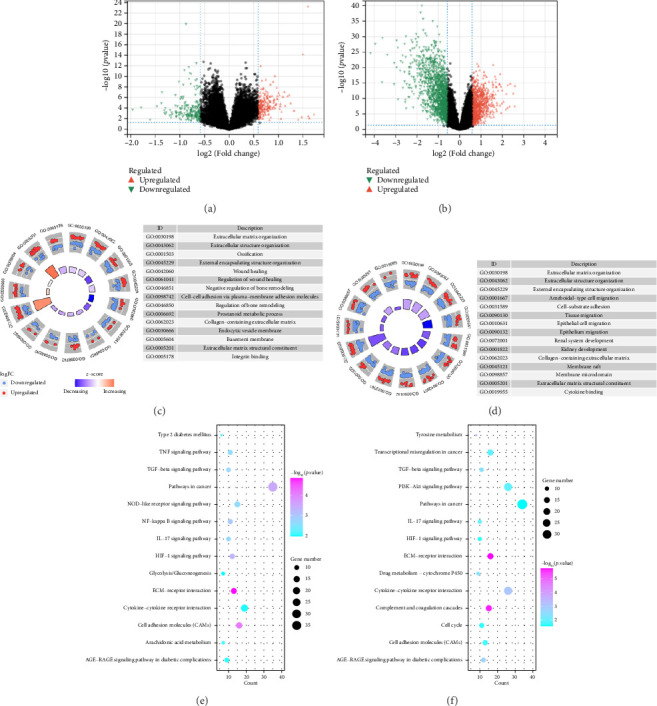
Identification and functional enrichment of DEGs: (a) volcano plot of differentially expressed genes in T2DM and normal pancreatic islets; (b) volcano plot of differentially expressed genes in T2DM and lung adenocarcinoma and normal lung tissue; (c) GO enrichment analysis for T2DM; (d) LUAD enrichment analysis for T2DM; (e) KEGG enrichment analysis of T2DM; (f) KEGG enrichment analysis. *Note:* DEGs: differentially expressed genes; T2DM: Type 2 diabetes mellitus; LUADL: lung adenocarcinoma; KEGG: Kyoto Encyclopedia of Genes and Genomes.

In the comparative genomic analysis, a study identified 651 DEGs when contrasting T2DM subjects with healthy individuals. Similarly, 799 DEGs were discerned in the comparison between LUAD samples and normal tissues. Analysis revealed that in T2DM, 340 DEGs were elevated in expression, while 311 showed reduced expression levels. In the case of LUAD, 216 genes were found to have higher expression levels, whereas 583 exhibited a decline (refer to Figures 1(a) and 1(b)). Additional pathways of interest included wound healing, its regulation, and cell–cell adhesion mediated through plasma membrane molecules. Notably, pathways involving the collagen-rich extracellular matrix (ECM), basement membrane composition, and structural constituents of the ECM were also prominent. GO enrichment analysis on LUAD revealed significant pathways, presented in Figures 1(c) and 1(d). Processes governing the organizational activities of external encapsulating structures were emphasized. The pathways also highlighted mechanisms of cell–substrate adhesion, along with the migration of tissues and specifically epithelial cells. Important pathways included those involving the collagen-rich ECM, its structural components, and cytokine interactions. T2DM and LUAD showed significant enrichment in ECM, epithelial adhesion, and cell movement.

KEGG analysis was performed on the DEGs between T2DM and LUAD, as illustrated in Figures 1(e) and 1(f). Notably, the tumor necrosis factor and transforming growth factor *β* (TGF-*β*) pathways were significantly altered. In addition, the research identified pathways common in cancer, NOD-like and NF-kappa B signaling pathways, along with IL-17 and HIF-1 signaling pathways. Metabolic pathways such as glycolysis and gluconeogenesis were also highlighted. Additional pathways that were notably enriched include interactions between ECM receptors, cytokine and cytokine receptors, as well as cell adhesion molecules. Moreover, pathways related to arachidonic acid metabolism and AGE–RAGE signaling, which are implicated in diabetic complications, were also identified among the other pathways. In the analysis of LUAD, specific pathways were notably altered, including tyrosine metabolism, transcriptional misregulation in cancer, TGF-*β* signaling, and PI3K–Akt signaling. Conversely, the study identified several enriched pathways such as those commonly associated with cancer, the IL-17 and HIF-1 signaling pathways, ECM–receptor interactions, and cytochrome P450 drug metabolism. Furthermore, interactions between cytokines and cytokine receptors, complement and coagulation cascades, and cell cycle regulation were significantly influenced. Additional pathways affected included cell adhesion molecules and the AGE–RAGE signaling pathway, which is relevant to diabetic complications, among others. KEGG enrichment analysis identified several overlapping pathways between T2DM and LUAD, highlighting shared biological processes. Highlighted pathways encompassed TGF-*β*, HIF-1, and IL-17 signaling, alongside the functions of cell adhesion molecules and the AGE–RAGE signaling mechanism. The convergence of these pathways in both conditions underscores a potential linkage, as established through both GO and KEGG analyses, enhancing the comprehension of the molecular interconnections between T2DM and LUAD.

### 3.2. Construction of Coexpression Networks and Analysis of Module Correlations Between T2DM and LUAD

**Figure 2:**
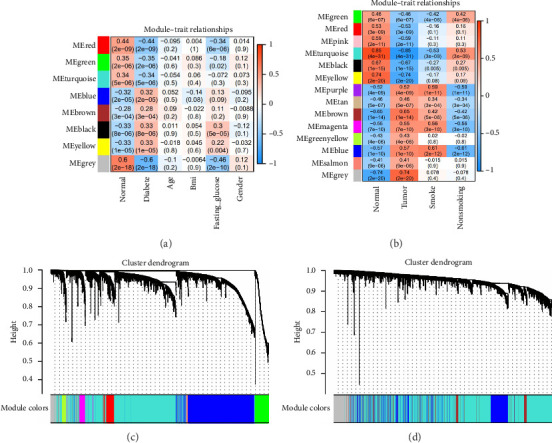
Construction of coexpression networks and analysis of module correlations between Type 2 diabetes mellitus (T2DM) and lung adenocarcinoma (LUAD): (a) heat map of association between gene modules and clinical shape in LUAD dataset GSE43458; (b) heat map of association between gene modules and clinical shape in T2DM dataset GSE76896; (c) cluster tree of T2DM dataset GSE76896; (d) cluster tree of LUAD dataset GSE43458.

To identify gene clusters with shared biological functions and establish their correlations with clinical traits, WGCNA was employed. For T2DM, eight distinct gene clusters, referred to as modules, were identified under a soft threshold of *β* = 7. In the case of LUAD, analysis using a soft threshold of *β* = 9 led to the identification of 14 modules. Gray modules, representing nonclustered genes, were excluded from the analysis. In T2DM, negative correlations were observed for genes in the red, green, and green–blue–green modules, while positive correlations were identified for genes within the blue, brown, black, and yellow modules (Figure 2(a)). In the LUAD analysis, negative correlations were observed for genes within the green, red, pink, blue–green, black, and yellow modules. Conversely, genes in the purple, brown–yellow, brown, magenta, yellow–green, blue, and light orange modules showed positive correlations with LUAD (Figure 2(b)). Using the DOSE package, the biological functions of each module were characterized through GO terms to explore the correlations between T2DM and LUAD. Notably, genes in the blue module of T2DM, which are primarily involved in cell development and metabolic processes, exhibited similarities to those in LUAD's brown module, as detailed in Figures 2(c) and 2(d). These modules are considered important in relation to T2DM and LUAD and should be managed through further research.

### 3.3. Screening of Key Genes With Clinical Significance

**Figure 3:**
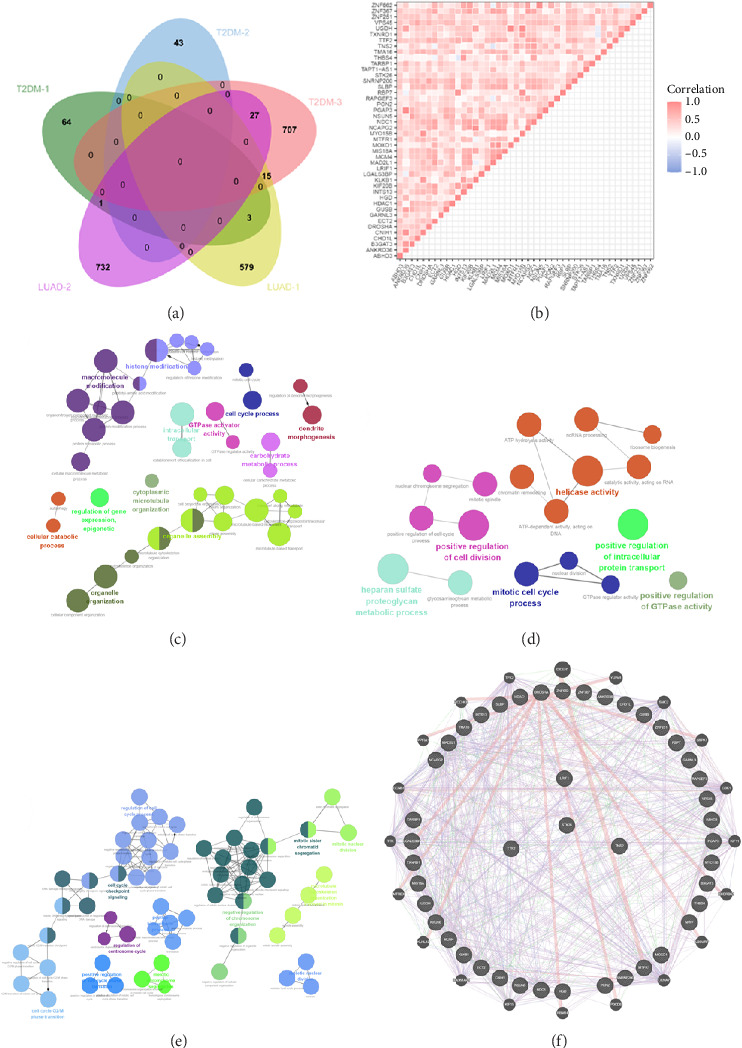
Screening of key genes with clinical significance: (a) shared genes between T2DM and LUAD; (b) correlation analysis of 44 shared genes in the TCGA database; (c) T2DM blue module; (d) ClueGO analysis of the LUAD brown module; (e) ClueGO analysis of the 44 shared genes; (f) protein interaction network of four shared genes. *Note:* T2DM: Type 2 diabetes mellitus; LUAD: lung adenocarcinoma.

In the “yellow,” “blue,” “black,” “gray,” and “brown” modules of positive association with T2DM and LUAD, 44 shared genes were positively associated with both T2DM and LUAD. The genes showed significant correlations with the pathogenesis of both diseases. Correlation analysis suggested that most of these shared genes showed significant mutual associations (Figures 3(a) and 3(b)).

Using ClueGO, we conducted GO analysis on genes located in the brown module for LUAD and the blue module for T2DM. The enrichment analysis of T2DM's blue module predominantly highlighted processes such as the cell cycle, intracellular transport, organelle assembly, and carbohydrate metabolism. In an effort to dissect the cellular mechanisms underlying the brown module of LUAD, a detailed enrichment analysis was conducted. This analysis revealed that this module plays a significant role in various phases of the cell cycle, notably in the progression, G2/M phase transitions, and checkpoint signaling. In addition, it pointed to the active role of microtubule structures during mitosis and the positive regulation of cell phase transitions. Further insights were pursued through a GO analysis with the aid of GlueGo, focusing on 44 genes shared between T2DM and LUAD. The functions of these genes span a broad spectrum of biological activities, such as modulating helicase activity, metabolizing heparan sulfate proteoglycans, orchestrating mitotic cell cycle processes, facilitating cell division, transporting proteins within cells, and regulating glutathione peroxidase activity, as illustrated in Figures 3(c), 3(d), and 3(e).

We used the GeneMANIA tool to perform protein network analysis of 44 shared genes, and these shared genes demonstrated close interactions in various biological functions, including ATPase activity; nuclear division during mitosis, which is intricately regulated and involves the organization of the microtubule cytoskeleton spindle formation; and chromosome alignment. In addition, control over the nuclear division is mediated through the activity of the cyclin-dependent protein kinase holoenzyme complex, which is integral to cellular cycle progression (Figure 3(f)).

### 3.4. Identification of Hub-Shared Genes and Their Prognostic Value in LUAD

**Figure 4:**
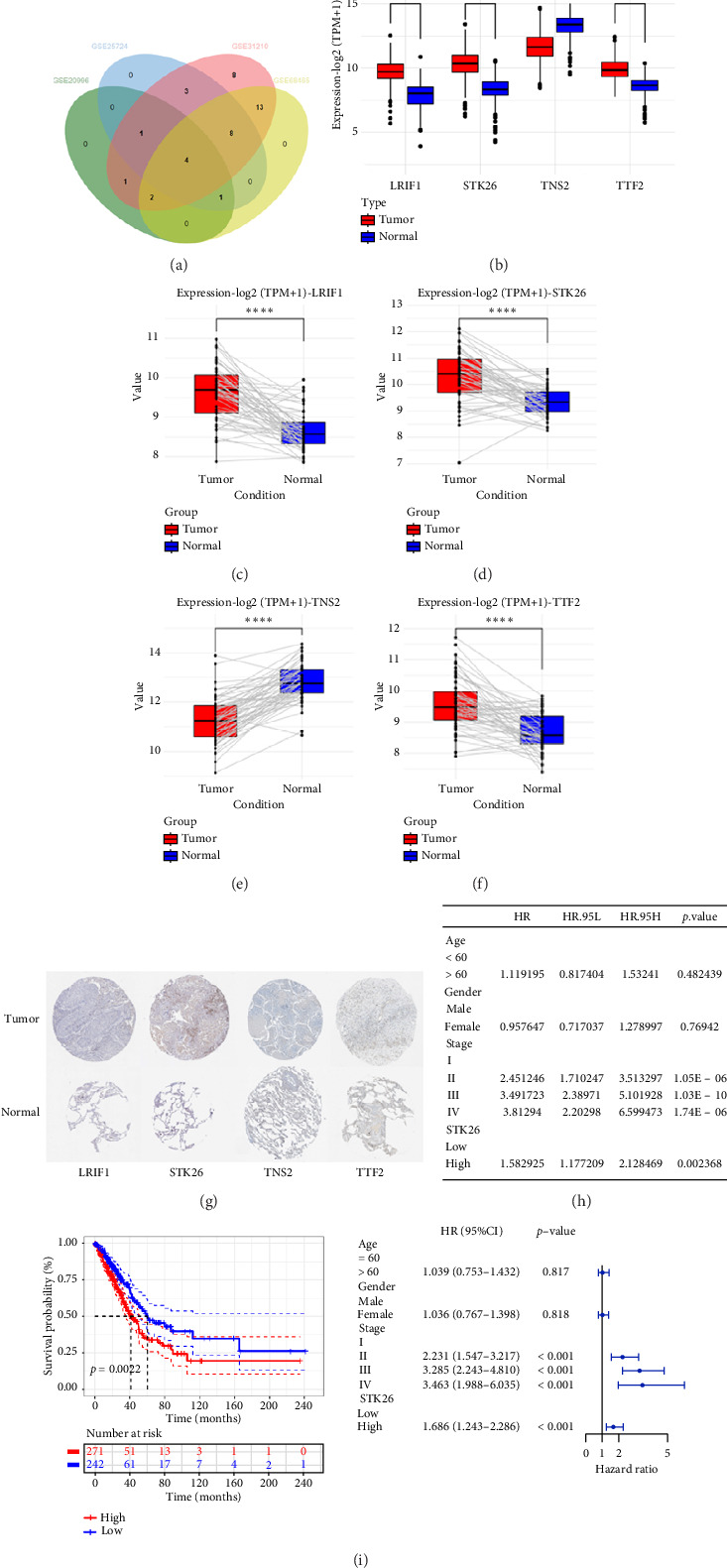
Identification of hub-shared genes and their prognostic value in LUAD: (a) Venn diagram of shared genes between T2DM and LUAD in the cohort was validated; (b–f) four hub-shared gene expression levels in tumor and normal tissues. ns, no significance; ^∗^*p*<0.05, ^∗∗^*p*<0.01, ^∗∗∗^*p*<0.001, and ^∗∗∗∗^*p*<0.0001; (g) protein expression levels of the four hub-shared genes in lung adenocarcinoma and normal lung tissues; (h) Kaplan–Meier survival curves based on median STK26 expression in TCGA–LUAD; (i) multivariate Cox regression forest plots based on median STK26 expression in TCGA–LUAD. *Note:* T2DM: Type 2 diabetes mellitus; LUAD: lung adenocarcinoma.

To substantiate our research, we utilized four validation cohorts, GSE25724, GSE20966, GSE31210, and GSE68465, comprising two cohorts each for T2DM and LUAD controls. Differential analyses were executed on case versus control samples within these cohorts. Subsequently, genes shared between the experimental cohorts (T2DM: GSE76896 and LUAD: GSE43458) were cross-referenced with the DEGs identified in the validation studies (Figure 4(a)). Finally, we identified four core shared genes, LRIF1, TNS2, TTF2, and STK26, indicating their significance in T2DM and LUAD. To explore the effects of these four genes on LUAD, we included LUAD data from the TCGA, GTEx, and Human Protein Atlas (HPA) databases. Expression levels for four pivotal genes shared between 513 LUAD tissues and 347 normal lung tissues were analyzed. Expression levels of the key genes TNS2, LRIF1, STK26, and TTF2 showed marked differences, exhibiting substantially elevated expression in tumor tissues versus normal tissues. In a detailed examination of 114 paired samples from the TCGA–LUAD cohort, significant variations were observed in the expression of LRIF1, STK26, TNS2, and TTF2 between tumor and normal tissues, with *p* values less than 0.001. These findings align with those from the combined TCGA–GTEx analysis, as illustrated in Figures 4(c), 4(d), 4(e), and 4(f). Immunohistochemical staining data from the HPA database was employed to verify the protein expression levels of the four central genes: LRIF1, TNS2, TTF2, and STK26. This analysis facilitated a comparison between their expressions in tumor tissues and normal tissues. An examination of STK26 showed a slight elevation in its expression in LUAD tissue when contrasted with normal lung tissue. An investigation into the prognostic significance of STK26 in LUAD was conducted by analyzing RNA-seq and clinical data from the TCGA–LUAD cohort. This analysis included categorizing patients within the TCGA–LUAD cohort into high and low STK26 expression groups, based on the median expression level as the division point. Kaplan–Meier survival analysis showed that higher STK26 expression was associated with reduced overall survival, reaching statistical significance (*p* = 0.0022). A univariate Cox regression analysis determined that survival outcomes were significantly affected by variables such as age, sex, cancer stage, and the level of STK26 expression. The hazard ratio (HR) for STK26 expression in LUAD was recorded at 1.58, with a 95% confidence interval (CI) ranging from 1.18 to 2.13, indicating a negative prognostic factor for these patients (*p*=0.002368). In LUAD, multivariate Cox regression analysis revealed that elevated STK26 expression significantly reduced overall survival, evidenced by a HR of 1.686 (*p* < 0.001). This positions STK26 as an independent prognostic marker, as illustrated in Figures 4(h) and 4(i).

### 3.5. The STK26 Gene Is Highly Associated With Metabolism-Related Pathways

Our investigation into STK26's influence on LUAD development incorporated a thorough approach with data sourced from the TCGA–LUAD cohort. We classified the patients into categories reflecting high and low expressions of STK26. Subsequently, we utilized GSEA to conduct enrichment analysis using the KEGG gene sets as the reference. In the investigation of STK26's biological roles, differential expression groups with high and low levels underwent enrichment analysis. Key metabolic processes including glycolysis–gluconeogenesis, oxidative phosphorylation, and the transformations between pentose and glucuronic acid, along with the pentose phosphate pathway, showed increased activity in the group with elevated STK26 expression.

Exploring STK26's function in biology, GSVA applied the hallmark gene set for analysis within the TCGA–LUAD cohort. This revealed that key biological processes such as glycolysis, hypoxia, MYC target activity, and oxidative phosphorylation were more pronounced in the group exhibiting higher STK26 expression compared to those with lower expression levels. Using KEGG as a reference, a GSVA analysis was conducted, linking the activity scores of gene sets with STK26 expression levels. The analysis established that elevated STK26 expression was significantly associated with the activation of key metabolic pathways. Notably, this includes enhanced activity in glycolysis–gluconeogenesis, oxidative phosphorylation, cell cycle processes, and the citric acid (TCA) cycle. This suggests that STK26 could influence the progression and onset of T2DM and LUAD through its regulatory impact on these metabolic pathways.

**Figure 5:**
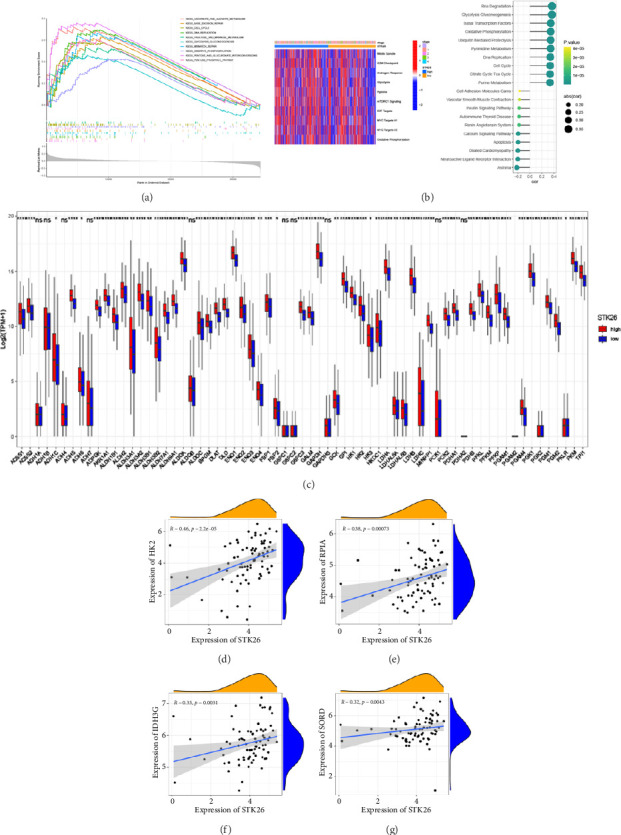
The STK26 gene is highly associated with metabolism-related pathways: (a) the top 10 pathways enriched by GSEA analysis in patients with high STK26 expression in TCGA–LUAD; (b) heatmap of GSVA with hallmark as a reference gene set and lollipop map of correlation between KEGG signaling pathway and STK26 expression; (c) box plot of the expression of genes involved in the glycolytic pathway in the STK26 high and low expression groups; (d–g) dot plot of correlation between STK26 expression and glycolytic pathway–related genes in lung adenocarcinoma cell lines. *Note:* KEGG: Kyoto Encyclopedia of Genes and Genomes; GSEA: gene set enrichment analysis; GSVA: gene set variation analysis.

Differential expression analysis showed that genes related to the glycolytic pathway were relatively highly expressed in the STK26 overexpression group (Figure 5).

### 3.6. Immunization Landscape Assessment

**Figure 6:**
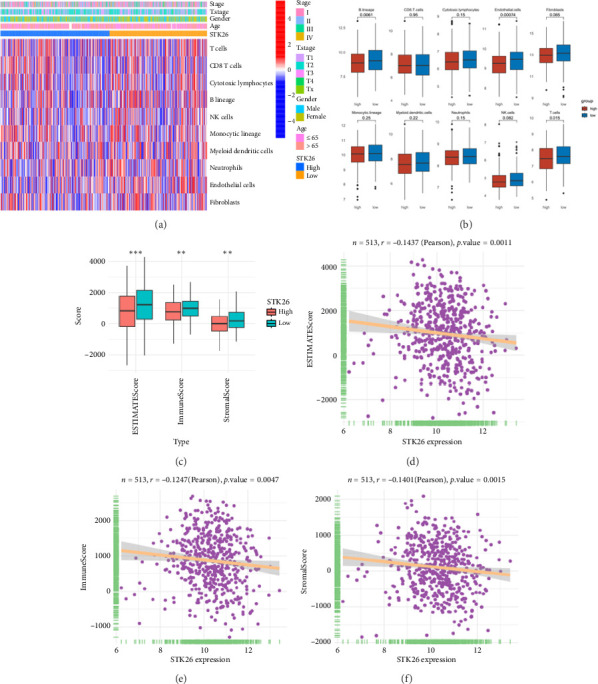
Immunization landscape assessment: (a) MCP–counter heatmap of STK26 high and low expression groups; (b) box plot of STK26 high and low expression groups; (c) the ESTIMATE algorithm box plot of STK26 high expression group and low expression group and the correlation between the expression of STK26 and ESTIMATEScore; (d) the ESTIMATE algorithm box plot of STK26 high expression group and low expression group and the correlation between the expression of STK26 and ImmuneScore; (e) the ESTIMATE algorithm box plot of STK26 high expression group and low expression group and the correlation between the expression of STK26 and StromalScore; (f) the ESTIMATE algorithm box plot of STK26 high expression group and low expression group and the correlation between the expression of STK26 and dot plot. ns, no significance; ^∗^*p*<0.05, ^∗∗^*p*<0.01, ^∗∗∗^*p*<0.001, and ^∗∗∗∗^*p*<0.0001.

The analysis employed the MCP–counter and ESTIMATE algorithms to evaluate how components of the tumor microenvironment correlate with STK26 expression. Results indicated that high STK26 expression correlates with reduced presence of immune cells, with significant decreases in T cells (*p*=0.015) and B lineage cells (*p*=0.0061), as detailed in Figures 6(a) and 6(b). The ESTIMATE algorithm quantifies stromal and immune cell prevalence in tumor tissues by leveraging differential gene expression data. It calculates the relative abundance of these cells through an analysis of genes that are differentially expressed between tumor and adjacent nontumor cells. This approach uses gene expression profiles from tumor samples to assess cellular infiltration levels. In concurrence with MCP–counter findings, analysis via the ESTIMATE algorithm demonstrated elevated scores for ESTIMATEScore, ImmuneScore, and StromalScore among samples with lower STK26 expression. Detailed statistical analysis showed significant negative correlations with STK26 expression levels: ESTIMATEScore (*r* = −0.1437, *p*=0.0011), ImmuneScore (*r* = −0.1247, *p*=0.0047), and StromalScore (*r* = −0.1401, *p*=0.0011), as illustrated in Figures 6(c), 6(d), and 6(e).

Figure 6 displays a series of visual comparisons between groups with high and low expressions of STK26. Part A shows a heatmap generated by the MCP–counter, and Part B displays a box plot comparing these groups. The ESTIMATE algorithm's influence is depicted in Part C through another box plot contrasting the two groups. Finally, Parts D, E, and F illustrate dot plots that map the correlations between STK26 expression and the ESTIMATEScore, ImmuneScore, and StromalScore, respectively.

### 3.7. STK26 Was Highly Expressed in LUAD Cell Lines Under HG Conditions *In Vitro*

**Figure 7:**
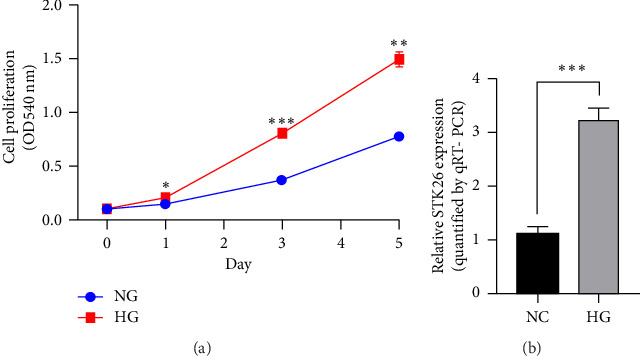
STK26 was highly expressed in LUAD cell lines under high glucose conditions *in vitro:* (a) CCK-8 growth curve in HG and NG groups; (b) STK26 mRNA expression in HG and NG groups. *Note:* LUAD: lung adenocarcinoma; HG: high glucose; NG: normal glucose. ns, no significance; ^∗^*p*<0.05, ^∗∗^*p*<0.01, ^∗∗∗^*p*<0.001, and ^∗∗∗∗^*p*<0.0001.

In an experiment to assess the impact of glucose levels on LUAD cell growth and STK26 expression, A549 cells were cultured in two different glucose concentrations: 25 mmol for the HG group and 5.5 mmol for the normal glucose (NG) group. The CCK-8 assay was conducted to measure cell growth, as depicted in Figure 7(a). Results showed that A549 cells proliferate more rapidly in the HG environment compared to the NG environment, with significant statistical differences in growth rates. In addition, to evaluate the effect on STK26 expression, the cells were also exposed separately to 25 and 5.5 μmol glucose concentrations in the respective HG and NG settings. After a 24 h culture period, RNA extraction was conducted, followed by quantification of STK26 expression through qRT-PCR analysis.  Figure 7(b) reveals that LUAD cells exhibit notably higher STK26 levels under HG conditions, comparing the HG group with the NG group, indicating that elevated glucose enhances STK26 expression.

Suppressing STK26 expression markedly curtailed the proliferation and migration of LUAD cells when exposed to HG environments during *in vitro* studies.

**Figure 8:**
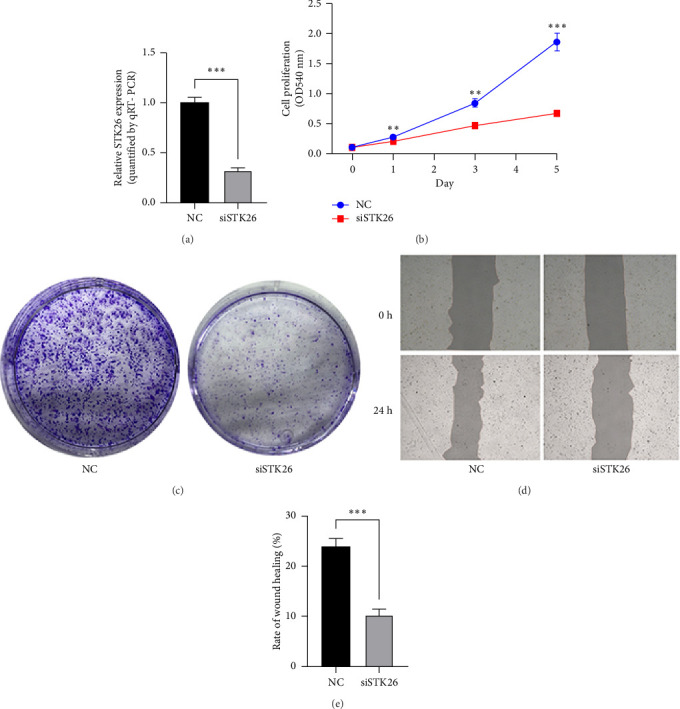
Suppressing STK26 expression markedly curtailed the proliferation and migration of LUAD cells when exposed to high glucose environments during *in vitro* studies: (a) STK26 mRNA expression after siRNA treatment in HG environment; (b) CCK-8 growth curve after siRNA treatment in HG environment; (c) colony formation after siRNA treatment in HG environment; (d, e), scratch test after siRNA treatment in HG environment. *Note:* LUAD: lung adenocarcinoma; HG: high glucose; NG: normal glucose. ns, no significance; ^∗^*p*<0.05, ^∗∗^*p*<0.01, ^∗∗∗^*p*<0.001, and ^∗∗∗∗^*p*<0.0001.

To verify the effect of STK26 on LUAD cells under HG conditions, an siRNA was designed to inhibit STK26 expression (Figure 8(a)). After achieving over 80% reduction in STK26 expression via qPCR, various tests such as the CCK-8 assay, colony formation, and scratch tests were conducted to determine the influence of STK26 on the proliferation and migration of lung cancer cells. Figures 8(b) and 8(c) illustrate these experiments. The data revealed a notable decline in the growth of LUAD cells following STK26 suppression. To assess the influence of STK26 on the migration of LUAD cells within a HG setting, a scratch assay was conducted. A549 cells, pretreated with siRNA, were evenly distributed across 6-well plates. Upon reaching confluence, a 200 μL pipette tip was used to create a linear scratch. The closure of this scratch was then monitored over a 24 h period, with results documented in Figures 8(d) and 8(e). With three replicative tests, the healing rates for the control and siRNA-treated groups measured 23.3% and 10.3%, respectively (*p* < 0.001). This significant disparity demonstrates that suppressing STK26 expression reduces both the proliferation and migration of LUAD cells under HG conditions.

## 4. Discussion

In recent decades, the prevalence of LUAD has overtaken squamous cell carcinoma, establishing it as the predominant form of lung cancer globally. This shift is evident in various regions, including the United States, Canada, Europe, and Japan, affecting both genders equally. Currently, LUAD represents half of all lung cancer cases and continues to rise in incidence. Historically, until the 1990s, squamous cell carcinoma had been more frequently diagnosed, especially among men.

Globally, more than 400 million individuals suffer from T2DM, classifying it as an epidemic [19], In China, this condition is notably prevalent, affecting 9.1% of the population and imposing substantial socioeconomic costs [20]. T2DM [21] is recognized as a significant risk factor for NSCLC and is associated with less favorable short-term surgical outcomes in these patients [22, 23]. The molecular links between LUAD and T2DM remain incompletely understood in existing research. The findings aim to enhance early detection, improve treatment options, and facilitate preventive strategies.

This research utilized bioinformatics approaches to pinpoint critical genes and modules that play a role in the progression of T2DM and the growth of LUAD. Global gene expression data from lung tissue can elucidate the pathogenesis of T2DM. Bioinformatic analysis revealed that several significant modules associated with both diseases exhibited genes concentrated in cellular communication and ECM organization. These genes also showed a high degree of enrichment in processes regulating the cell cycle and cell division, among others. The overlap of genes and regulatory mechanisms in LUAD and diabetes mellitus (DM) suggests a shared pathogenesis between these conditions.

Contemporary research reveals that pathways activated by insulin and IGF-1, such as PI3K/Akt kinase and Ras/MAP kinase, facilitate the proliferation and spread of tumor cells.TGF-*β* has long been identified with its intensive involvement in early embryonic development and organogenesis, immune supervision, tissue repair, and adult homeostasis. Overexpressed TGF-*β* causes a plethora of metabolic disorders and dysfunction, and promotes epithelial–mesenchymal transition (EMT) and excessive deposition of ECM. It also leads to NF-B activation and an increase in HIF-1 mRNA expression by NF-B. NF-B is activated, which induces the expression of proinflammatory genes and increases HIF-1 expression. Elevated insulin levels may generate hydrogen peroxide, inducing oxidative stress and exacerbating damage through mutations in tumor-related genes. Moreover, the chronic inflammation frequently seen in diabetic conditions is likely to enhance cancer cell proliferation and metastasis.

Differential gene expression analysis across four validated cohorts confirmed the involvement of 44 genes shared between LUAD and T2DM. Among these, STK26 emerges as a critical gene potentially influential in the development of both LUAD and T2DM.

STK26 belongs to the serine–threonine kinase family. This gene serves as a critical regulator across various signaling pathways closely linked to tumor progression and initiation, such as the P13K/Akt kinase, Ras/MAP kinase, and IGF-1-activated pathways [24, 25]. Current research revealed that LUAD samples exhibit higher levels of STK26 expression compared to normal tissue. Furthermore, a notable reduction in overall survival rates was observed in patients exhibiting STK26 overexpression compared to their counterparts without such overexpression.

Recent studies highlight the significant influence of the tumor immune microenvironment (TIME) on the evolution and advancement of cancer [26, 27]. Our study identifies a notable negative correlation between STK26 expression and the abundance of different immune cells including follicular helper T cells (Tfh), cytotoxic cells, and dendritic cells (DCs) in individuals with PC. Tfh cells, categorized within the CD4+ T cell subset, essential for the formation of germinal centers, antibody maturation, and the maintenance of humoral memory in both autoimmune diseases and cancer settings [28], when they infiltrate, serve a protective role in breast and colorectal cancers [29]. Cytotoxic T cells, also known as CD8+ cells, alongside DCs, are recognized for their tumor-opposing properties. Cancer cells can escape detection and destruction by the immune system by upregulating ligands that activate inhibitory checkpoints. This process effectively suppresses the activation of cytotoxic T lymphocytes (CTLs), which are essential for targeting and eliminating malignant cells [30, 31].

This investigation was constrained by a few significant limitations. The use of public databases, which frequently feature clinical data of limited scope and potential bias, might have influenced the results. Moreover, to clarify the shared regulatory mechanisms influencing endodermal cell destiny in LUAD and T2DM, additional *in vitro* studies are essential. It is also imperative to conduct future clinical trials to ascertain the prognostic accuracy of STK26 in patients with T2DM and LUAD, given the insufficiency of the existing evidence.

However, there are still some limitations in this study. First, the limited clinical information available in public databases may lead to bias in WGCNA results. Second, more *in vitro* experiments are needed to further demonstrate the molecular mechanisms and signaling pathways common to LUAD and T2DM. Further validation is needed in future clinical trials. In conclusion, this study suggests common genetic signatures sufficient to elucidate the possible mechanisms of LUAD and T2DM and identifies STK26 as a key gene.

The findings confirm that STK26 is a common gene influencing both LUAD and T2DM, with a notable impact on the TIME.

## 5. Conclusion

In summary, this study revealed that the STK26 gene may play a biological role in LUAD by influencing metabolic pathways, especially the glycolysis pathway, and the high expression of the STK26 gene suggests poor immune infiltration in LUAD. In the presence of HG levels, LUAD cells showed increased proliferation and migration, which was associated with increased STK26 expression. In this case, targeted inhibition of STK26 significantly reduced the expansion and spread of these malignant cells. The STK26 gene was identified as an immune-related biomarker and potential therapeutic target in LUAD patients with T2DM.

## Data Availability

The datasets used and/or analyzed during the current study were publicly available from the GEO database in the following website: https://www.ncbi.nlm.nih.gov/geo/.
